# Influence of Group on Individual Subject Maps in SPM Voxel Based Morphometry

**DOI:** 10.3389/fnins.2016.00522

**Published:** 2016-12-02

**Authors:** Andrew M. Michael, Eli Evans, Gregory J. Moore

**Affiliations:** ^1^Autism & Developmental Medicine Institute, Geisinger Health SystemLewisburg, PA, USA; ^2^Institute for Advanced Applications, Geisinger Health SystemDanville, PA, USA; ^3^Department of Radiology, Geisinger Health SystemDanville, PA, USA

**Keywords:** structural MRI preprocessing, SPM DARTEL, SPM VBM, spatial normalization, influence of group

## Abstract

Voxel based morphometry (VBM) is a widely utilized neuroimaging technique for spatially normalizing brain structural MRI (sMRI) onto a common template. The DARTEL technique of VBM takes into account the spatial intensity distribution of sMRIs to construct a study specific group template. The group template is then used to create final individual normalized tissue maps (FINTM) for each subject in the group. In this study, we investigate the effect of group on FINTM, i.e., we evaluate the variability of a constant subject's FINTM when other subjects in the group are iteratively changed. We examine this variability under the following scenarios: (1) when the demographics of the iterative groups are similar, (2) when the average age of the iterative groups is increased, and (3) when the number of subjects with a brain disorder (here we use subjects with autism) is increased. Our results show that when subject demographics of the group remains similar the mean standard deviation (*SD*) of FINTM gray matter (GM) of the constant subject was around 0.01. As the average age of the group is increased, mean *SD* of GM increased to around 0.03 and at certain brain locations variability was as high as 0.23. A similar increase in variability was observed when the number of autism subjects in the group was increased where mean *SD* was around 0.02. Further, we find that autism vs. control GM differences are in the range of −0.05 to +0.05 for more than 97% of the voxels and note that the magnitude of the differences are comparable to GM variability. Finally, we report that opting not to modulate during normalization or increasing the size of the smoothing kernel can decrease FINTM variability but at the loss of subject-specific features. Based on the findings of this study, we outline precautions that should be considered by investigators to reduce the impact of group on FINTM and thereby derive more meaningful group differences when comparing two cohorts of subjects.

## Introduction

Voxel Based Morphometry (VBM) in SPM is an industry leading tool applied to preprocess structural MRI (sMRI) of the brain (Ashburner and Friston, 2000). VBM registers sMRIs of different subjects to a common template through spatial normalization. Spatial normalization enables users to make inter-subject comparisons between brains that have different geometric shapes and topography in their native space. Group level analyses are made by examining brain tissue map concentrations that are derived from VBM. VBM has the option of computing a study specific group template through Diffeomorphic Anatomical Registration using Exponentiated Lie algebra (DARTEL; Ashburner, 2007). Each subject's sMRI is then normalized to the study specific template before performing group analyses. Here, we investigate the effect of group composition on final individual normalized tissue maps (FINTM) that are constructed from the group template.

VBM has been widely applied in neuroimaging studies and in diverse subject populations. For example, VBM has been used to track cerebellar degeneration in Alzheimer's disease (Colloby and Taylor, 2014) and to map gray matter atrophy in Lewy body dementia (Watson et al., 2012). VBM has been utilized to detect group differences in brain structure in autism spectrum disorder (Abell et al., 1999; Calderoni et al., 2012; Riva et al., 2013), pervasive developmental disorder (Kosaka et al., 2010), epilepsy (Keller and Roberts, 2008), and schizophrenia (Honea et al., 2005; Asami et al., 2012). Imaging genetics studies have also made use of VBM DARTEL to study 22q11.2 deletion in children (Campbell et al., 2006). Because VBM provides effective normalization techniques, it has become a popular method for making group level volumetric comparisons of brain tissues even in heterogeneous populations.

Our motivation to examine the accuracy of VBM DARTEL's study specific template is the outcome of a serendipitous finding while preprocessing a set of sMRIs that accidentally included a “phantom” (a calibration device) image. The image of the phantom, when preprocessed using VBM with other brain sMRIs, went through normalization successfully. After preprocessing, we observed that the normalized image of the non-brain phantom had taken on “brain-like” topography. This made us question how the inclusion of this phantom may have influenced the FINTMs of other brain sMRIs. We removed the phantom and re-ran all of the sMRIs and found that the FINTMs this time were different from their counterparts in the previous run. This inspired us to remove more brain sMRIs and examine the effect on the remaining subjects. Again we saw changes in FINTM voxel values. From this finding we were led to examine the phenomenon of FINTMs being altered as a function of the other subjects present in the group. In this project, we examine the effect of group on FINTM using a set of structured experiments.

Previous studies have examined the effect of variations in demographics and image acquisition methods on VBM results. VBM differences based on scanner (Focke et al., 2011), and how these scanner-based differences were affected by the group gender composition (Takao et al., 2014) have been reported previously. Takao et al. reported that gender composition (ratio of males to females) between different scanner sites can result in varying FINTMs, but it is unclear if the variability in FINTM was caused by scanner differences or gender composition. Results of the above studies were based on multisite data with heterogeneous subject demographics and their focus was not on evaluating the effects of group composition on FINTM.

Other VBM studies have looked at the importance of selecting appropriate VBM parameters. VBM parameters that have been investigated include size of smoothing kernel, type of atlas used, modulation, and the number of subjects in the group. García-Vázquez et al. (2008) examined the effects of different smoothing kernel sizes and group size on the ability to detect atrophy. A later study recommended a smoothing kernel of 6 mm for groups with 50 participants and 8–10 mm for groups with 25 participants (Shen and Sterr, 2013). They concluded that parameter selection changed the significance of the voxels while using the same group of subjects (Shen and Sterr, 2013). In addition, the effects of modulation were demonstrated in Williams Syndrome where the shape of the hypothalamus and orbitofrontal gray matter regions were dependent on whether the data was modulated or not (Eckert et al., 2006). The above studies indicate the importance of paying close attention to the parameters used while preprocessing sMRIs using VBM.

However, previous studies do not address how overall subject composition of the group affects FINTMs when FINTMs are constructed using a study specific template. Previous studies do not answer the question of “can the FINTM of a particular subject change if that subject is preprocessed with different groups of subjects?” In this study, we address this question by investigating how FINTMs of subjects that are held constant (henceforth *constant subject*) change when they are preprocessed with varying groups of subject compositions. The answer to the above question is important as alterations of FINTM as a function of the group can affect post processing analyses that use FINTM, especially analyses of group differences between two cohorts of subjects.

Using real sMRI data and a set of systematic experiments we test the influence of group on the FINTM of constant subjects. In Experiment 1, we investigate the effects of changing groups when the demographics of the groups are not substantially different. Experiment 1 serves as a baseline or “gold standard” to quantify the variability of FINTM when group compositions between runs are not considerably different demographically. We then compare the variability of Experiment 1 with FINTM changes when group compositions are more heterogeneous. In Experiment 2, we investigate the effects of changing the group by widening the age range of the subject pool at each preprocessing run. In Experiment 3, we examine the effects of altering the ratio of the number of autism spectrum disorder (ASD) patients and typically developing controls (TDC). This experiment is similar to Experiment 2, however, group composition is varied with respect to diagnosis rather than age. In Experiment 4, we compare ASD vs. TDC FINTM differences of all ASD and TDC subjects in our sample and through Experiment 4 we aim to compare the variability of a constant subject's FINTM with ASD vs. TDC differences. Experiments 5 and 6 examine the effect of modulation and spatial smoothing on FINTMs of constant subjects.

## Methods

### VBM-DARTEL

The final outputs of VBM are spatially normalized subject specific tissue maps. We refer to these maps as FINTMs. VBM segments sMRI into three primary tissue concentration maps: gray matter (GM), white matter (WM), and cerebrospinal fluid (CSF). VBM performs this segmentation by first affine transforming each sMRI to the Montreal Neuroimaging Institute (MNI) template, and then using prior probability maps to determine the probability of each voxel belonging to one of the tissue maps. Each segmented tissue map has voxel values between 0 and 1 and represents tissue concentration (Ashburner and Friston, 2000). For example, a voxel value of 0.7 in the GM tissue map indicates that the probability or likelihood of GM concentration at that voxel is 70%. In the DARTEL pipeline of VBM, after brain sMRIs are segmented into different tissue classes, a group specific normalization template is created for each tissue type (Ashburner, 2007). The study specific group template is created using an iterative process. It is generated by creating a mean image from the individual maps, and then matching the individual maps to this mean template. After the individual images are morphed to match the group template, a new template is generated as a mean of the new images. This process is repeated for a set number of runs creating a progressively crisper template. The GM and WM images are then normalized to the final template. As such, the final template that is created using the above averaging mechanism is a function of the tissue maps that were used to create it. A voxel concentration modulation is performed (except in Experiment 5) to preserve the amount of tissue content. During modulation each voxel concentration is scaled by the amount of expansion or contraction of that voxel that occurred during spatial normalization. The normalized images are then smoothed using a Gaussian kernel to increase the signal to noise ratio by decreasing the effects of small errors in registration (Ashburner, 2007). Gaussian kernel smoothing also approximates tissue voxel concentrations to a normal distribution (Ashburner, 2007).

### Subjects

MRI images used in this study were downloaded from the Autism Brain Imaging Data Exchange (ABIDE), a multisite online database of brain images from subjects with autism and typically developing controls (http://fcon_1000.projects.nitrc.org/indi/abide/; Di Martino et al., 2014). For all experiments we used T1-weighted sMRIs of male subjects scanned on a Siemens Magnetom Verio 3T scanner at the New York University (NYU) Langone Medical Center. SMRIs used in this study had the following pulse sequence parameters: TR = 2530 ms, TE = 3.25 ms, bandwidth = 200 Hz/Px, flip angle = 7^0^, echo spacing = 7.4 ms, slice thickness = 1.33 mm, voxel size = 1.3 × 1.0 × 1.3 mm, field of view = 256 mm, and base resolution = 256 mm.

Subjects were selected from a single site (NYU) to reduce the effects of scanner and acquisition variability present across sites of the ABIDE dataset. This removes the possibility of FINTM variability that can be attributed to scanner and imaging protocol differences affecting the group template and focuses on FINTM variability due to just subject variability in group composition. In addition, to remove variability added by gender, only male subjects were picked for the experiments presented in this study. Among the ABIDE sites, NYU was chosen since it had the largest number of subjects. Evaluation of image quality was performed through visual inspection of each image. Images with motion (ghosting and smearing), susceptibility and homogeneity artifacts were removed from further analysis. ASD and TDC subjects were pairwise chosen to match age as close as possible. Our pool of subjects consisted of 24 TDC and 24 ASD subjects (age range TDC: 10.5–14.8 years, ASD: 10.5–14.7 years; mean age ± *SD* TDC: 12.52 ± 1.45 years, for ASD: 12.54 ± 1.45 years; *t*-val = 0.06; *p*-val = 0.95). Subjects for Experiment 1, 3, and 4 were drawn from the above pool of subjects. In Experiment 2, because the effects of increasing age are investigated, in addition to the above pool, TDCs between 11 and 29 years were also included. Experiments 5 and 6 are extensions of Experiment 2. Further details on subject ages are provided in the Experiments Section.

### Preprocessing

Images were preprocessed according to the guidelines, parameters and templates recommended in the VBM tutorial (Ashburner, 2010) except for the smoothing kernel, which was kept at 6 mm to improve the accuracy of DARTEL for registration and normalization. A 6 mm kernel is recommended to more accurately detect changes (Shen and Sterr, 2013). The 8 mm smoothing kernel recommended by the VBM tutorial was used in Experiment 6 to compare the effects of increasing age group on FINTM with varying smoothing kernels. Images were segmented into GM, WM, and CSF tissue maps using prior probability maps included with SPM8 for tissue classification. The maps were then spatially normalized, modulated (except in Experiment 5), and then smoothed.

### Experiments

The main aim of this paper is to investigate how constant subjects' FINTMs vary when the groups in which they are preprocessed are iteratively changed. Our analyses are focused on changes of GM FINTM. We focused only on FINTM voxel locations with GM concentrations above a threshold of 0.1. As voxels locations with <0.1 GM concentrations have <10% GM content those voxels were not included for further analysis. In each experiment we iteratively and randomly subsampled a group of subjects. In each group run, three subjects were held constant and an evaluation of the variability across runs was done via the standard deviation (*SD*) of GM concentration. We report the mean and *SD* of GM concentration across all runs for each of the three constant subjects. We also report the overall distribution of GM *SD* and display the *SD* of GM concentration on a brain map.

#### Experiment 1: FINTM variability when preprocessed in groups with similar demographics

The purpose of Experiment 1 is to investigate how FINTMs of constant subjects change when the group demographics are similar between runs. We randomly picked three TDCs from our pool of 24 TDCs and these three TDCs were kept as constant subjects between runs. In each run we randomly picked an additional 12 TDCs from the remaining 21 TDCs, added these 12 TDCs to the three constant TDCs and preprocessed a total number of 15 TDCs. The 12 subjects that changed with each run had an age range of 10.46–14.79 years with a mean age of 12.52 years. Age information for each of the 20 runs is provided in Table 1. Two sample *t*-tests between subject ages across all combinations of runs were mostly (>96%) statistically insignificant.

**Table 1:** **Age data for Experiment 1: Mean, Standard Deviation, and age range for the group of 15 typically developing controls (TDC) for each of the 20 runs**.

***N* = 15**	**Age; μ ± σ (range)**		**Age; μ ± σ (range)**		**Age; μ ± σ (range)**		**Age; μ ± σ (range)**
Run 1	13.1 ± 1.5 (10.7–14.7)	Run 6	12.4 ± 1.4 (10.5–14.7)	Run 11	12.4 ± 1.4 (10.5–14.7)	Run 16	13.1 ± 1.2 (10.5–14.7)
Run 2	12.7 ± 1.3 (10.5–14.7)	Run 7	12.6 ± 1.3 (10.5–14.7)	Run 12	12.5 ± 1.5 (10.5–14.7)	Run 17	12.5 ± 1.3 (10.7–14.5)
Run 3	12.5 ± 1.5 (10.5–14.7)	Run 8	12.9 ± 1.3 (10.5–14.7	Run 13	13.1 ± 1.3 (10.8–14.7)	Run 18	12.2 ± 1.6 (10.5–14.5)
Run 4	12.6 ± 1.4 (10.5–14.7)	Run 9	12.7 ± 1.4 (10.5–14.7)	Run 14	12.5 ± 1.4 (10.7–14.7)	Run 19	13.2 ± 1.3 (10.7–14.7)
Run 5	13.1 ± 1.4 (10.7–14.7)	Run 10	11.8 ± 1.2 (10.5–14.2)	Run 15	12.5 ± 1.5 (10.5–14.7)	Run 20	13.2 ± 1.4 (10.7–14.7)

*Twelve subjects were chosen randomly from a pool of 21 TDC subjects while three were held constant across the 20 runs. Two sample t-test p-values were mostly insignificant*.

#### Experiment 2: FINTM variability when preprocessed in groups with increasing age

The purpose of Experiment 2 is to check for changes in constant subjects' FINTM while the mean age of the group is increased. Here our null hypothesis H0 is that “compared to the variability of Experiment 1 there is no significant increase in variability.” We repeatedly processed sMRI, holding the youngest three TDCs constant and changing the 12 TDCs for 15 different runs. Only 15 runs were used in this experiment due to the limited number of TDCs available at higher ages. The 12 TDCs that were changed in each run were selected using a sliding window across age, with the younger three TDCs from the group of 12 being removed and the next three oldest being added. The age range of Run 1 was 10.46–12.81 years and of Run 15 was 10.46–29.02 years. Two sample *t*-tests between the subject age of Run 1 and the subject age of all other runs (except Run 2) were statistically significant. Note that for this Experiment we deliberately increased the mean age of each group. Age information for the 15 runs and *P*-values of two sample *t*-test difference from Run 1 are provided in Table 2.

**Table 2:** **Age data for Experiment 2, 5, and 6: Mean, Standard Deviation, and age range for the 15 runs**.

***N* = 15**	**Age; μ ± σ (range)**		**Age; μ ± σ (range)**		**Age; μ ± σ (range)**
Run 1	11.6 ± 0.8 (10.5–12.8)	Run 6	14.1 ± 1.9 (10.5–16.1)	Run 11	17.1 ± 3.5 (10.5–20.5)
			*P* < 1*E* − 4		*P* < 1*E* − 4
Run 2	12.1 ± 1.0 (10.5–13.7)	Run 7	14.5 ± 2.1 (10.5–16.5)	Run 12	18.1 ± 4.0 (10.5–22.5)
	*P* = 0.14		*P* < 1*E* − 4		*P* < 1*E* − 4
Run 3	12.6 ± 1.3 (10.5–14.4)	Run 8	15.0 ± 2.3 (10.5–16.9)	Run 13	19.1 ± 4.4 (10.5–23.1)
	*P* = 0.01		*P* < 1*E* − 4		*P* < 1*E* − 4
Run 4	13.1 ± 1.5 (10.5–14.8)	Run 9	15.5 ± 2.6 (10.5–19.1)	Run 14	20.0 ± 4.9 (10.5–25.3)
	*P* = 2*E* − 3		*P* < 1*E* − 4		*P* < 1*E* − 4
Run 5	13.7 ± 1.7 (10.5–15.4)	Run 10	16.3 ± 3.1 (10.5–20.2)	Run 15	21.5 ± 5.9 (10.5–29.0)
	*P* = 2*E* − 4		*P* < 1*E* − 4		*P* < 1*E* − 4

*In each Run, 12 typically developing controls (TDC) were randomly chosen from 21 TDC subjects while the three youngest TDC subjects were held constant. At each Run the mean age of the group is incremented. Two sample t-test P-values of age between Run 1 and other Runs are presented*.

#### Experiment 3: FINTM variability when preprocessed in groups with increasing ASD/TDC ratio

In this Experiment, we examine how ASD/TDC ratio affects the FINTMs. Here again our null hypothesis H0 is that “compared to the variability of Experiment 1 there is no significant increase in variability.” We repeated a process similar to Experiments 1 and 2 by holding three randomly chosen TDCs constant and changing 21 other subjects at each run. For the first run, of the 21 subjects not held constant, 20 were TDC and 1 ASD. For each subsequent run, one TDC was randomly removed and one age matched ASD was added from the original ASD pool of 24 subjects. Age information for the 20 runs are provided in Table 3. Age differences for all of the runs were statistically insignificant.

**Table 3:** **Age data for Experiment 3: Mean, Standard Deviation, and age range of 24 subjects for the 20 runs**.

***N* = 24**	**Age; μ ± σ (range)**		**Age; μ ± σ (range)**		**Age; μ ± σ (range)**		**Age; μ ± σ (range)**
Run 1	12.6 ± 1.4 (10.5–14.8)	Run 6	12.4 ± 1.4 (10.5–14.8)	Run 11	12.7 ± 1.5 (10.5–14.8)	Run 16	12.6 ± 1.4 (10.5–14.7)
Run 2	12.3 ± 1.5 (10.5–14.8)	Run 7	12.4 ± 1.5 (10.5–14.7)	Run 12	12.7 ± 1.5 (10.5–14.7)	Run 17	12.6 ± 1.4 (10.5–14.7)
Run 3	12.8 ± 1.5 (10.5–14.7)	Run 8	12.5 ± 1.6 (10.5–14.8)	Run 13	12.6 ± 1.3 (10.5–14.7)	Run 18	12.6 ± 1.4 (10.5–14.7)
Run 4	12.5 ± 1.6 (10.5–14.8)	Run 9	12.5 ± 1.4 (10.7–14.7)	Run 14	12.6 ± 1.4 (10.5–14.7)	Run 19	12.7 ± 1.4 (10.5–14.7)
Run 5	12.7 ± 1.6 (10.5–14.8)	Run 10	12.5 ± 1.3 (10.5–14.7)	Run 15	12.8 ± 1.3 (10.5–14.7)	Run 20	12.6 ± 1.4 (10.5–14.7)

*Three TDC subjects were chosen randomly to be constant across runs. At each run, TDC/ASD ratio is changed to include one fewer TDC and one more ASD subject. Two sample t-test p-values were mostly insignificant*.

#### Experiment 4: distribution of ASD vs. TDC GM differences

In Experiment 4, we examine the distribution of ASD vs. TDC GM differences to compare the magnitude of these differences with those found in FINTM variability for Experiments 1–3. For example, questions such as “are ASD vs. TDC differences in the range of FINTM variability of Experiments 1–3?” and “what is the maximum value of the ASD vs. TDC difference and how does it compare with the maximum value of FINTM variability of Experiments 1–3?” will be answered through this Experiment. To compare ASD vs. TDC GM differences we used all 24 ASDs and 24 TDCs.

#### Experiment 5: FINTM variability with no modulation

Experiments 1–4 were performed using modulation after the normalization step of preprocessing. In Experiment 5, we perform preprocessing using the same parameters and subjects as Experiment 2 but without modulation. Age data for Experiment 5 is provided in Table 2.

#### Experiment 6: FINTM variability with different smoothing kernels

Experiments 1–5 were performed using a 6 mm smoothing kernel during preprocessing. In Experiment 6, we repeat Experiment 2 with smoothing kernels of 4 and 8 mm. Here, we explore the effect of smoothing kernel on FINTM variability across the following three smoothing kernel sizes: 4, 6 mm (Experiment 2), and 8 mm. Age data for Experiment 6 is provided in Table 2.

## Results

### Experiment 1: FINTM variability when preprocessed in groups with similar demographics

**Figure 1:**
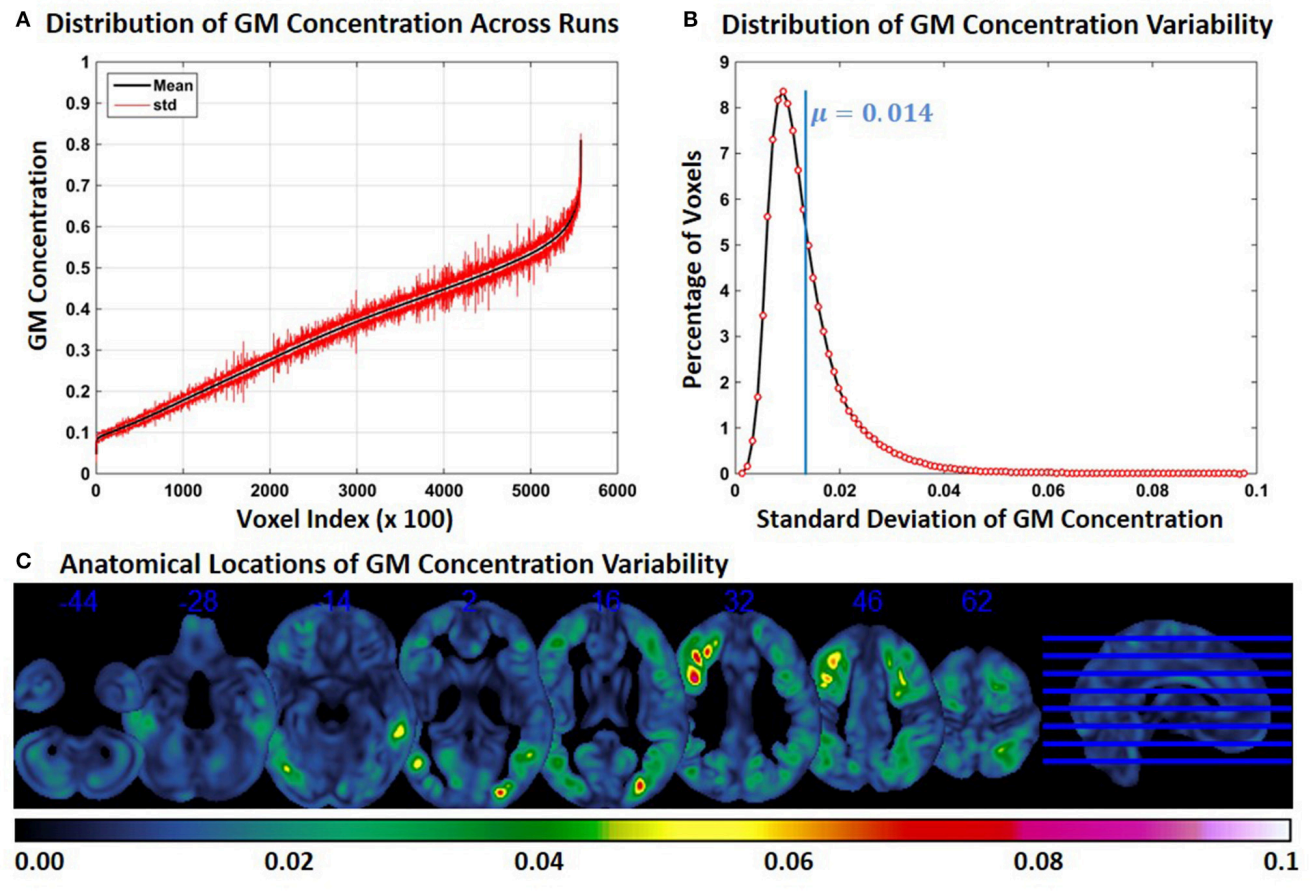
**FINTM variability when preprocessed in groups with similar demographics (Experiment 1). (A)** GM concentration variability of a constant subject across multiple runs; mean value is depicted in black and standard deviation in red. Mean GM concentrations are in ascending order and voxel indices are at intervals of 100. **(B)** The overall distribution of GM concentration variability. **(C)** Brain map of GM concentration variability (standard deviation).

For each constant subject, we computed the mean and standard deviation (across runs) of GM concentration at each FINTM voxel location. Figure 1A shows the mean (black) and standard deviation (red) of GM concentration across the whole brain of constant subject 1. For better visualization, GM concentrations were first ordered by mean GM concentration. Voxel values across the whole brain (at every 100 voxels) are presented in Figure 1A. In Figure 1B the distribution of GM *SD* is presented as a histogram. The histogram of *SD* indicates that about 8% of the voxels had a *SD* of around 0.01 and that the mean *SD* of all voxels is 0.014. The median of the *SD* distribution was 0.011 indicating that for more than 50% of the voxels the *SD* of gray matter concentration was above 0.011. The maximum *SD* of gray matter concentrations was 0.1. In Figure 1C, we present the anatomical locations of voxel variability. *SD*s of voxel variability are rendered on axial slices of the brain and color-coded as indicated by the colorbar. Figure 1C indicates that at certain brain locations *SD* of voxel clusters can be as high as 0.07. Most voxels exhibiting higher *SD* were not randomly scattered throughout the brain but present as clusters. GM concentration variability for the other two constant subjects are presented as Supplementary Figures 1, 2 and the nature of results were similar. The mean value of *SD* for both subjects was 0.015; and as in constant subject 1, the variability of GM concentrations were spatially clustered.

### Experiment 2: FINTM variability when preprocessed in groups with increasing age

**Figure 2:**
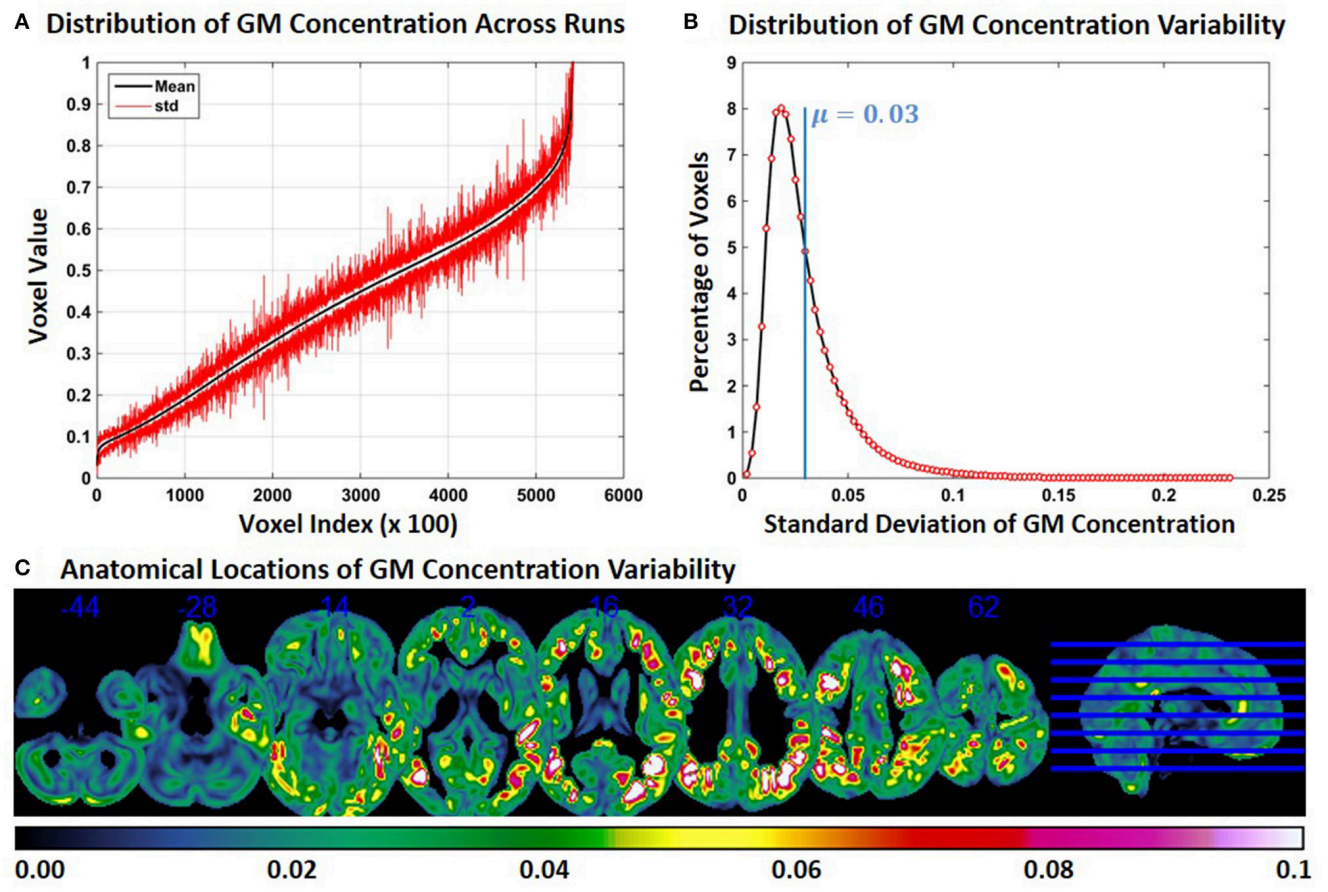
**FINTM variability when preprocessed in groups with increasing age (Experiment 2). (A)** GM concentration variability of a constant subject across multiple runs; mean value is depicted in black and standard deviation in red. Mean GM concentrations are in ascending order and voxel indices are at intervals of 100. **(B)** The overall distribution of GM concentration variability. **(C)** Brain map of GM concentration variability (standard deviation).

Experiment 2 showed an exacerbation of the distribution of FINTM voxel values when the constant subjects were processed with groups with increasing age range. This result is presented in Figure 2 for constant subject 1. Figure 2A indicates that the variability for GM concentrations across runs is higher than the variability in Experiment 1. Higher variability is observed at all voxel locations in the brain. In Figure 2B, we present the distribution of GM concentration variability. The histogram of GM *SD* indicates that about 8% of the voxels had a *SD* of around 0.02 and that the mean *SD* of all voxels is 0.03. The median of the *SD* distribution was 0.024 indicating that for more than 50% of the voxels the *SD* of gray matter concentration was above 0.024. The maximum *SD* of GM concentrations was 0.233. Figure 2C follows the same scheme as Figure 1C. At multiple brain locations the cluster centers had a GM concentration variability as high as 0.2. In Figure 2C the variability of GM concentrations are higher than that of Experiment 1. In Figure 2C we note that, compared to Figure 1C, more brain regions exhibit higher variability (slices viewed is kept constant between Figure 1C and Figure 2C). There is some overlap between the regions of Figure 1C and Figure 2C; but most of the high variability regions in Figure 2C were not present in Figure 1C. We checked for the consistency of this result by examining the other two constant subjects and noted similar findings. The mean value of *SD* for constant subjects 2 and 3 were 0.026 and 0.029, respectively. GM concentration variability maps of constant subjects 2 and 3 are provided as Supplementary Figures 3, 4 respectively.

### Experiment 3: FINTM variability when preprocessed in groups with increasing ASD/TDC ratio

**Figure 3:**
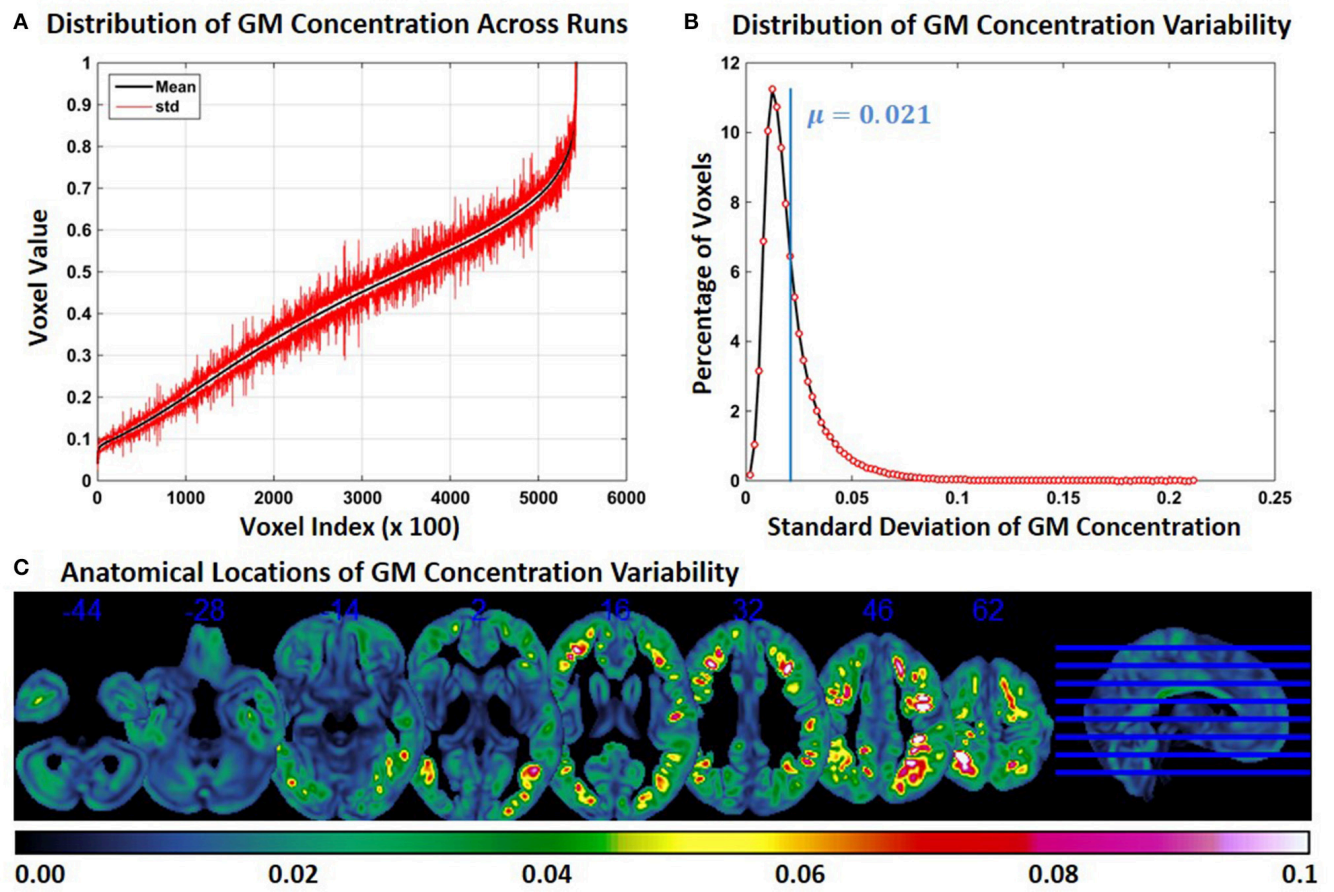
**FINTM variability when preprocessed in groups with increasing ASD/TDC ratio (Experiment 3). (A)** GM concentration variability of a constant subject across multiple runs; mean value is depicted in black and standard deviation in red. Mean GM concentrations are in ascending order and voxel indices are at intervals of 100. **(B)** The overall distribution of GM concentration variability. **(C)** Brain map of GM concentration variability (standard deviation).

Compared to Experiment 1, GM concentration variability in Experiment 3 was higher, but variability was less compared to Experiment 2. Figure 3A shows the *SD* of GM concentration for constant subject 1 for brain voxels across the whole brain. In Figure 3B the distribution of GM concentration variability is presented as a histogram. The histogram of the *SD* indicates that about 11% of the voxels had a *SD* of around 0.012 and that the mean *SD* of all voxels is 0.021. The median of the *SD* distribution was 0.017 indicating that for more than 50% of the voxels the *SD* of gray matter concentration was above 0.017. The maximum *SD* of gray matter concentrations was 0.21. At multiple brain locations, the cluster centers had a GM concentration variability as high as 0.15. Figure 3C shows brain locations with corresponding GM concentration variability. Compared to Figure 2C, regions with variability are less widespread, but more widespread than those in Figure 1C. There is some overlap between Figures 1C, 2C, 3C, but most of the regions do not overlap. GM concentration variability maps of constant subject 2 and 3 are provided as Supplementary Figures 5 and 6.

### Experiment 4: distribution of TDC vs. ASD GM concentration differences

**Figure 4:**
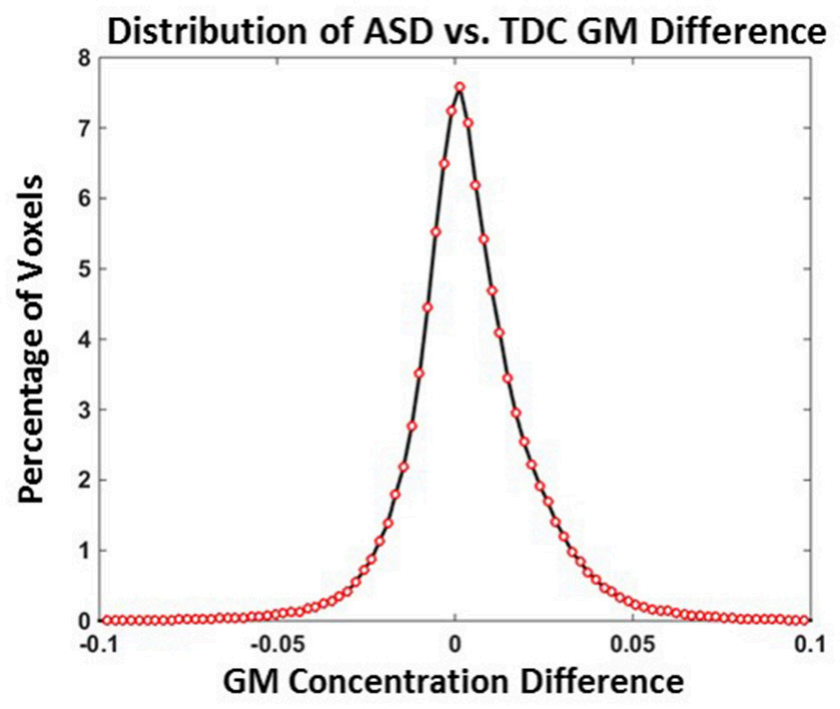
**Distribution of ASD vs. TDC GM difference (Experiment 4)**. ASD vs. TDC GM concentration differences *N*_*ASD*_ = *N*_*TDC*_ = 24.

Distribution of TDC vs. ASD GM concentration differences when all 24 TDCs and 24 ASDs are used is provided in Figure 4 and the mean GM difference is around 0. The number of voxels with a GM concentration difference >0.1 or <−0.1 were <0.04%. The number of voxels with a GM concentration difference >0.05 or <−0.05 were <2.3%. The above results indicate that TDC vs. ASD GM differences are small and predominantly present in the −0.1 to 0.1 range.

### Experiment 5: FINTM variability with no modulation

**Figure 5:**
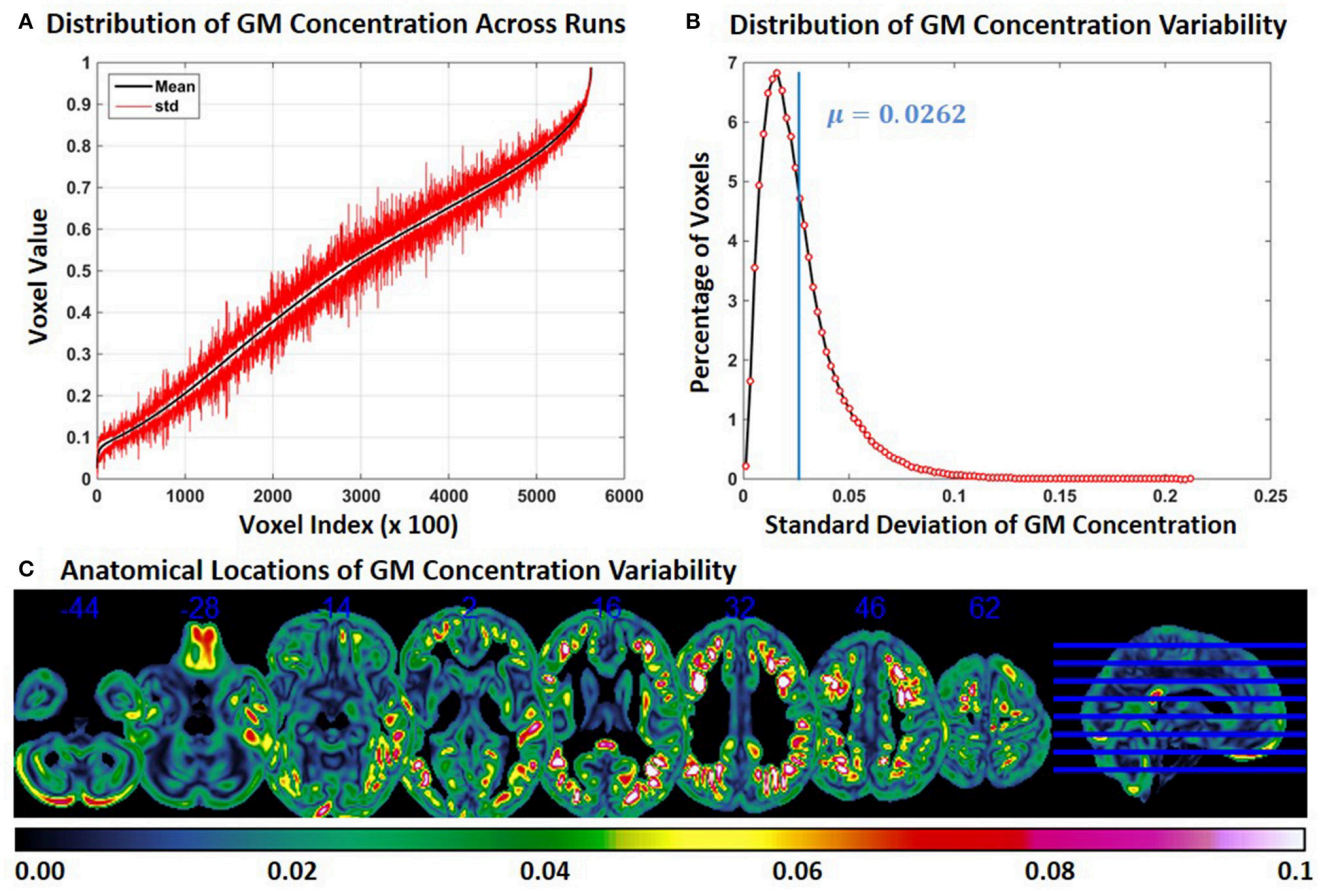
**FINTM variability with no modulation (Experiment 5). (A)** GM concentration variability of a constant subject across multiple runs; mean value is depicted in black and standard deviation in red. Mean GM concentrations are in ascending order and voxel indices are at intervals of 100. **(B)** The overall distribution of GM concentration variability. **(C)** Brain map of GM concentration variability (standard deviation).

In Experiment 5, we repeat Experiment 2 without modulating the GM concentrations. Compared to Experiment 2, the variability of GM concentration of constant subject 1 was slightly lower in Experiment 5. Mean and *SD* of GM concentration, distribution of GM concentration and brain locations of GM *SD* are presented in Figure 5. The mean value of GM concentration *SD* was 0.0262. When Figure 2C and Figure 5C are compared, we note that GM variability occurs at similar brain locations, but the magnitude of *SD* is generally higher in Figure 2C indicating that modulation can increase GM concentration variability.

### Experiment 6: FINTM variability with different smoothing kernels

**Figure 6:**
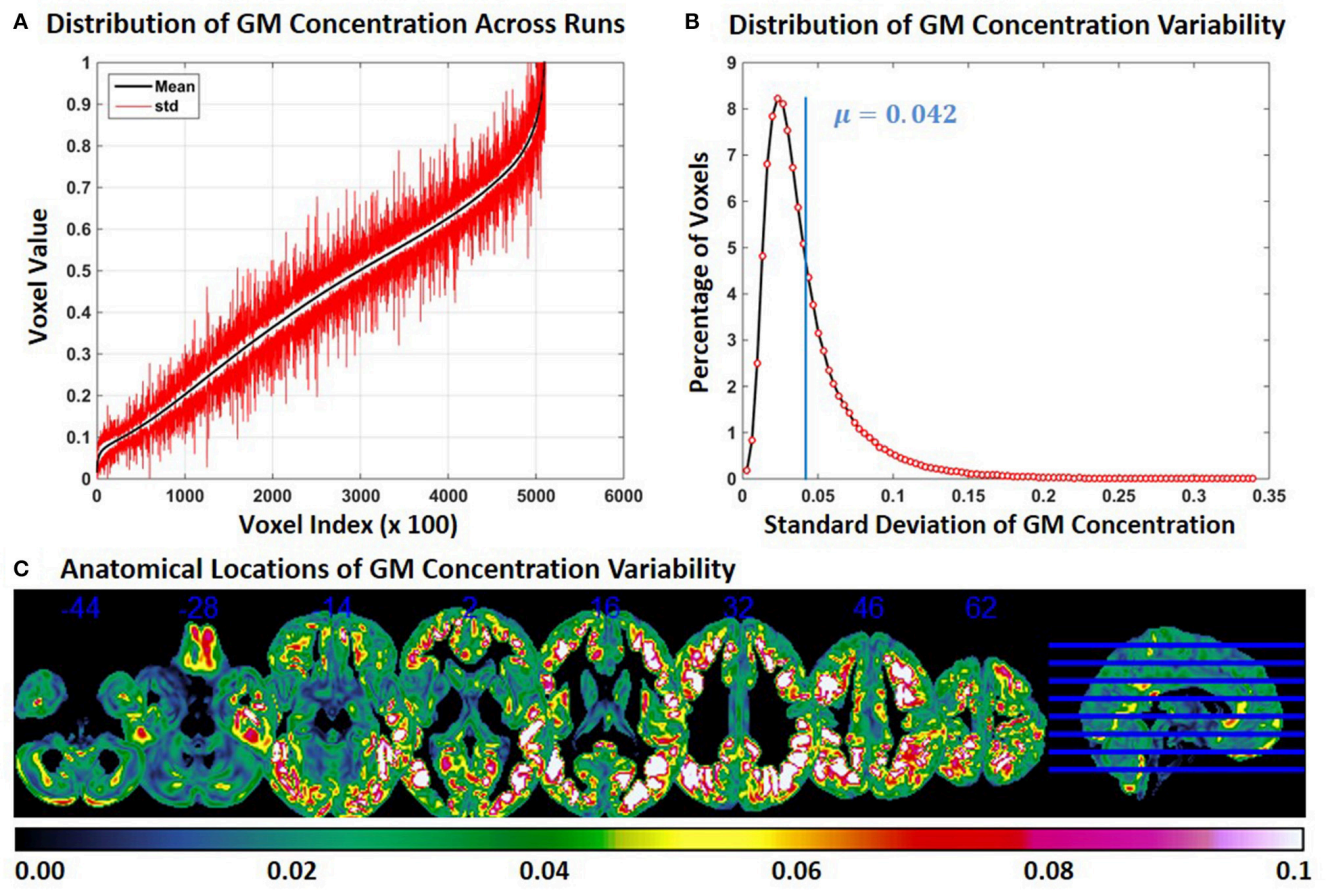
**FINTM variability with 4 mm smoothing (Experiment 6). (A)** GM concentration variability of a constant subject across multiple runs; mean value is depicted in black and standard deviation in red. Mean GM concentrations are in ascending order and voxel indices are at intervals of 100. **(B)** The overall distribution of GM concentration variability. **(C)** Brain map of GM concentration variability (standard deviation).

**Figure 7:**
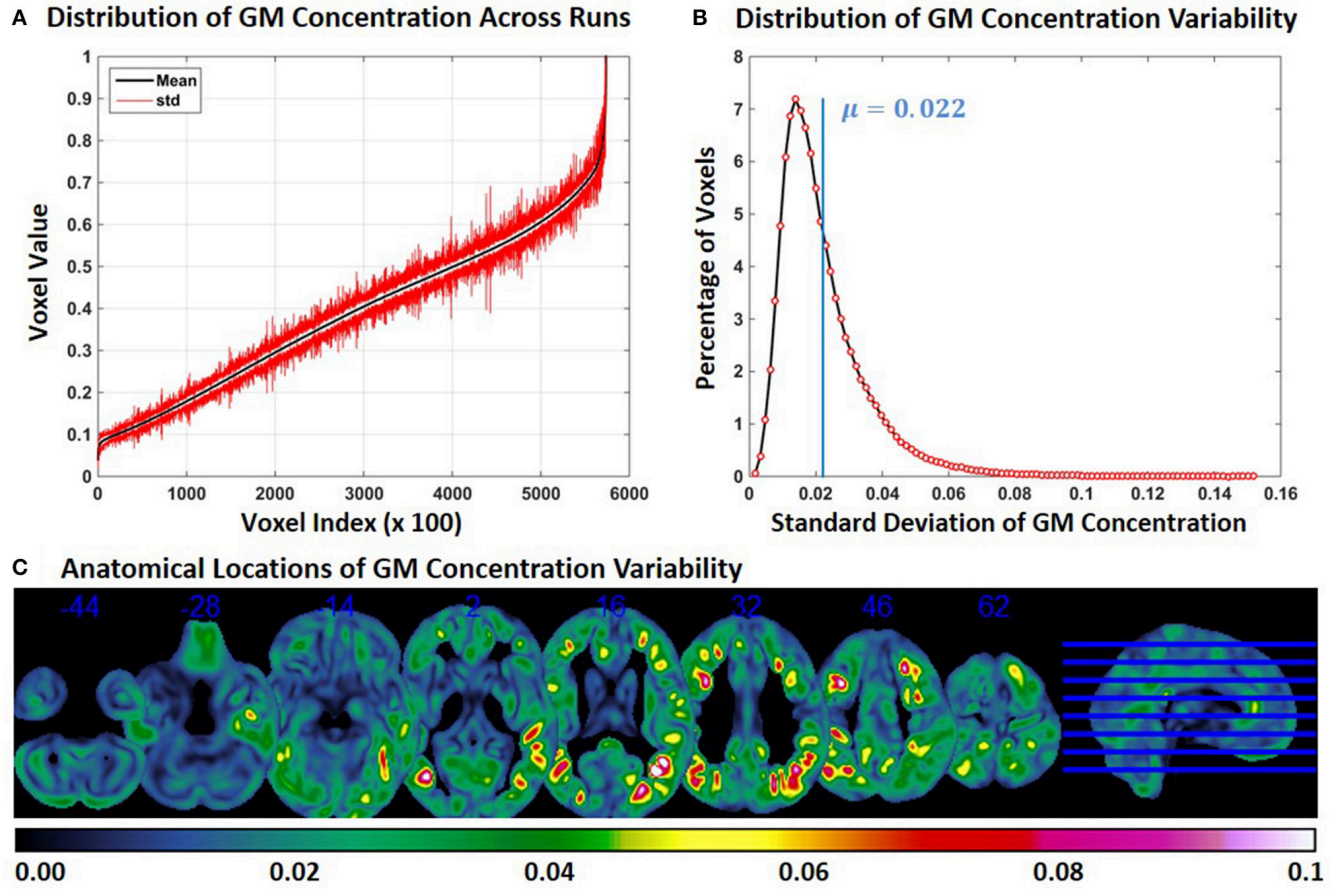
**FINTM variability with 8 mm smoothing (Experiment 6). (A)** GM concentration variability of a constant subject across multiple runs; mean value is depicted in black and standard deviation in red. Mean GM concentrations are in ascending order and voxel indices are at intervals of 100. **(B)** The overall distribution of GM concentration variability. **(C)** Brain map of GM concentration variability (standard deviation).

In Experiment 6, we repeat Experiment 2 with 4 and 8 mm smoothing kernels (Experiment 2 was performed with a smoothing kernel of 6 mm). Compared to Experiment 2, variability of GM concentration of constant subject 1 was higher with 4 mm smoothing and lower with 8 mm smoothing. Mean and *SD* of GM concentration, distribution of GM concentration and brain locations of GM *SD* for 4 and 8 mm smoothing are presented in Figures 6, 7 respectively. The mean value of GM concentration *SD* for 4 and 8 mm smoothing kernels was 0.042 and 0.022, respectively. Here, again, the GM variability occurred at similar brain locations, for 4 mm (Figure 6C), 6 mm (Figure 2C), and 8 mm (Figure 7C) smoothing, but the *SD* of GM concentration variability decreased with higher smoothing kernel size.

## Discussion

### Interpretation of results

In Table 4, we present a summary of results for FINTM GM variability across all experiments. Of the first three experiments, Experiment 2 (increasing age) showed the highest variability, followed by Experiment 3 (increasing ASD). The least variability was observed in Experiment 1 where subject demographics were similar across runs. We also note that not modulating during VBM and increasing the size of the smoothing kernel can reduce FINTM variability.

**Table 4:** **Summary of FINTM GM variability results across the different experiments**.

**Experiment #**	**Constant subject #1**			**Constant subject #2**	**Constant subject #3**
	**Mean**	**Median**	**Max**	**Mean**	**Mean**
1: Similar demographics	0.014	0.011	0.1	0.015	0.015
2: Increasing age	0.030	0.024	0.23	0.026	0.029
3: Increasing ASD	0.021	0.017	0.21	0.019	0.019
5: No modulation	0.026	0.021	0.21		
6: 4 mm smoothing	0.042	0.033	0.34		
8 mm smoothing	0.022	0.019	0.15		

*Experiments 1, 2, and 3 were performed with modulation and with a 6 mm smoothing kernel. Experiments 5 and 6 use the same experimental setup as Experiment 3 but uses no modulation and different smoothing kernels, respectively*.

In Experiment 1, we note small changes in the FINTMs of constant subjects. In Experiment 1, multiple runs were performed with different subsets of subjects with similar demographics (narrow age range, all male, all TDC). From our results, we conclude that the group templates between the runs may not have been very different. The brain regions in Figure 1C with high GM *SD* were not randomly scattered but occurred in clusters. This indicates that there may be a consistent set of regions that are prone to higher group variability. This result needs further investigation. In Figure 1C, GM variability for all brain regions were calculated using the *SD* across all runs for constant subject 1. In Supplementary Figures 1, 2 we present the brain regions of high variability for constant subjects 2 and 3, respectively. A visual comparison of regions of high GM concentration variability across the three subjects reveals that there are both overlapping and non-overlapping regions. This indicates that the high variability is caused by features that are both common across the group and unique to the individual subject.

When the age range of the preprocessing group was incremented in Experiment 2, the variability of GM concentration increased (Figure 2). In Experiment 3, a similar effect was observed when we changed the group to include a higher number of ASDs and fewer TDCs (Figure 3). In each run of Experiment 3, although the number of ASDs was increased, the average group age of each run was not changed. In both Experiments 2 and 3, the GM variability of the three constant subjects were on a similar scale. These results indicate that, compared to Experiment 1, FINTM variability increases with age and ASD/TDC ratio with age having a higher impact than ASD/TDC ratio. We speculate that this variability can be attributed to the variability of sMRI between different age groups and sMRI differences between ASD and TDC. Overall our results show that the process of normalizing a sMRI to a group template can result in variations on subjects' FINTMs that are dependent on the group of subjects that was used to construct the FINTMs. The increase in variability that accompanied the increase in age range and ASD/TDC ratio provides evidence that care should be taken when selecting subjects as wider group disparities can add higher group deviations to FINTM. In addition, in Experiments 1–3, we observed that the variability was clustered and not randomly scattered as individual voxels. This result indicates that variability is present in large brain regions (on the order of dozens of 1 mm^3^ voxels) that are not consistent across subjects. Most of the FINTM variability was evident in the gyri and sulci of the cortex. Each cortical folding pattern represented by gyri and sulci is likely unique to each individual (Van Essen and Dierker, 2007) and these subject specific patterns can be lost when FINTMs are derived from group templates.

In Experiment 4, the distribution of TDC vs. ASD GM concentration differences were explored using all 24 ASD and 24 TDC subjects. A histogram of GM group differences for Experiment 4 provides evidence that more than 99% of TDC vs. ASD differences GM concentrations are present between −0.1 and +0.1 and more than 97% of differences are present between −0.05 and +0.05. In Experiment 2, we observed that the mean *SD* of GM variability is 0.03 and can be as high as 0.233. The above values indicate that the *SD* of GM variability of a constant subject is comparable to the GM differences between two groups for >97% of the voxels. This result shows that voxels that are found to have significant ASD vs. TDC differences may be impacted by group variability.

In Experiment 5, we compared unmodulated FINTMs to modulated while age was increased across runs as in Experiment 3. We found that the variability after modulation is higher compared to no modulation. Modulation uses warping measures to correct for changes in volume based on the group template, and it is an additional processing step that will cause FINTM to be influenced by the group. Most VBM studies compare volumetric differences between groups and in such cases modulation is a necessary step to preserve volumetric changes that occur during the normalization step. But if comparing local GM volumes is not the main research question, our results show that using unmodulated GM concentrations is better as they will be less affected by the group.

Increasing the smoothing kernel decreased variability between runs for constant subject 1 in Experiment 6, but at the expense of losing subject specific features. Finding the optimal size of the smoothing kernel will always be important and dependent on the set of data being used. Ashburner recommends choosing a smoothing kernel that reflects the size of the regions of interest being explored (Ashburner, 2007), which is well-suited for a hypothesis based approach.

### Suggestions for VBM users

Several trends in variability patterns were evident in our results and can be used to guide a user for better VBM practices. Increasing the group age range as well as increasing the ratio of cases to controls can significantly alter the amount of variability observed in the FINTMs. When designing a study, it is necessary to take both of these factors into account. Care must be taken when preprocessing a group of controls with a larger group of cases. It will be important to assess if the variability of control FINTMs increases with a higher number of cases. If this is true we suggest that cases vs. controls comparisons be made on an equal number of samples in separate runs with subgroups of cases in each run. The consistency of the results across the different runs can then be compared. This also applies when the number of controls are larger than cases. Wider age ranges are also an important consideration as this can affect the FINTMs in a similar manner. These differences are not trivial and can produce inconsistent results. GM regions that appear in a subject when preprocessed with one group of individuals may be different in shape or even completely absent for this same subject when preprocessed with a different group of individuals. Our results indicate that demographic factors like age range and the ratio of patients to controls in a preprocessing group should be carefully considered. For these reasons careful attention to the selection of groups in studies is paramount. We suggest that users carefully match subjects based on demographics. Our results indicate that caution should be taken when drawing conclusions based on sMRIs that have been normalized to a group template using VBM DARTEL, especially when the age range of the subjects is large. In such cases, the final normalized images can differ depending on the group that the subject was processed with. To ascertain result robustness we suggest users perform permutation testing using sub groups of subjects.

### Limitations of our experiments

We performed all of our experiments using SPM8 using the recommendations of Ashburner (Ashburner et al., 2012) with data that was publically available. We did not verify our results with SPM12. SPM12 offers various improvements to normalization and segmentation, but according to SPM12 release notes (http://www.fil.ion.ucl.ac.uk/spm/software/spm12) the DARTEL process of normalizing to an averaged group template was not updated in SPM12. As such we speculate that our results will hold true with SPM12, but to confirm this result we plan to use SPM12 in future studies. Another limitation of this study is the small sample size which precludes measuring the variability of interest as a function of sample size.

## Conclusions

We demonstrate that the GM maps of individual subjects constructed by SPM's VBM DARTEL process can change with respect to the group in which they are preprocessed. These effects can be exacerbated when subject age is varied across preprocessing groups. We demonstrate that differences in age range, ratio of cases to controls, smoothing kernel size, and whether data is modulated or unmodulated can all affect the variability of the final GM maps. When using VBM DARTEL to preprocess brain sMRI data sets, all the above factors should be taken into consideration to reduce the confounding influence of group characteristics. These finding suggest that investigators should perform iterative permutation tests in imaging studies to improve the robustness of the results. This will ensure that the observed findings are not a byproduct of preprocessing artifacts.

## Author contributions

AM designed the experiments, developed metrics of evaluations, supervised EE and wrote and edited the manuscript with EE. EE implemented the experiments and wrote the manuscript under the guidance of AM. GM supervised the project, provided suggestions to AM and edited the manuscript.

### Conflict of interest statement

The authors declare that the research was conducted in the absence of any commercial or financial relationships that could be construed as a potential conflict of interest.
