# Supplementary material for: Influence of Group on Individual Subject Maps in SPM Voxel Based Morphometry
Source: Front Neurosci. 2016 Dec 2;10:522. doi: 10.3389/fnins.2016.00522 (PMC5134364; doi:10.3389/fnins.2016.00522)
Supplement: Supplementary file 1 [file DataSheet1.docx]

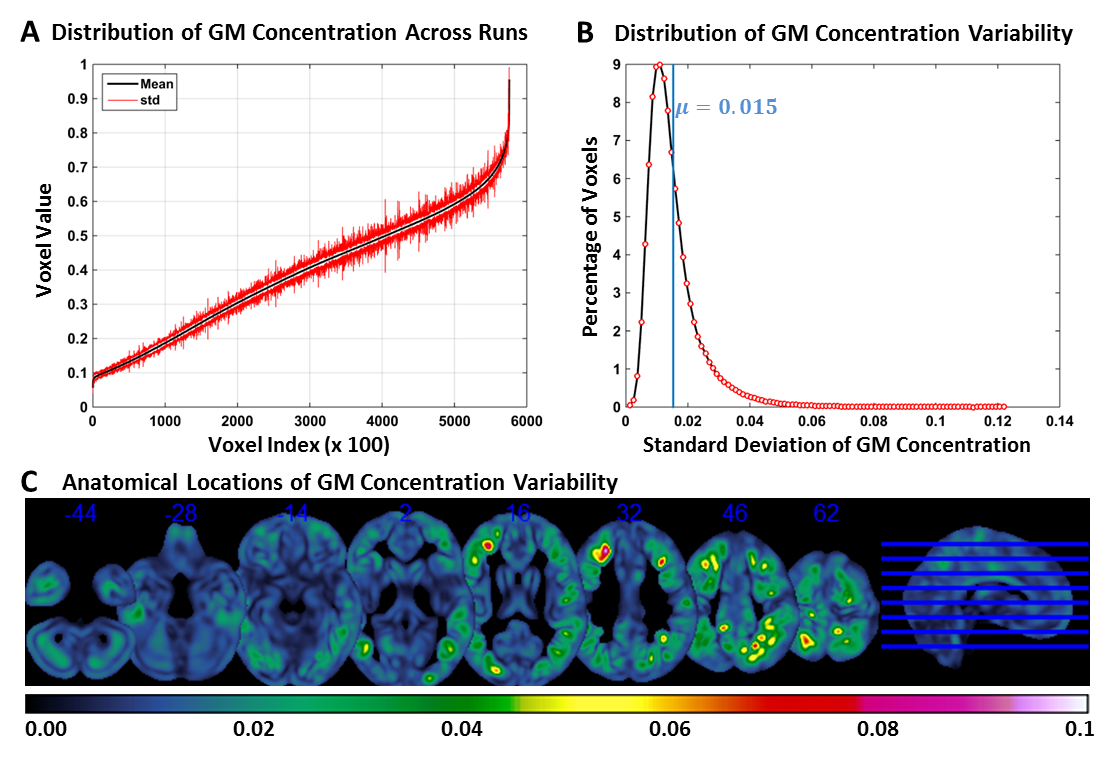


**Supp. Figure 1:** FINTM variability when preprocessed in groups with similar demographics (Experiment 1, constant subject 2).


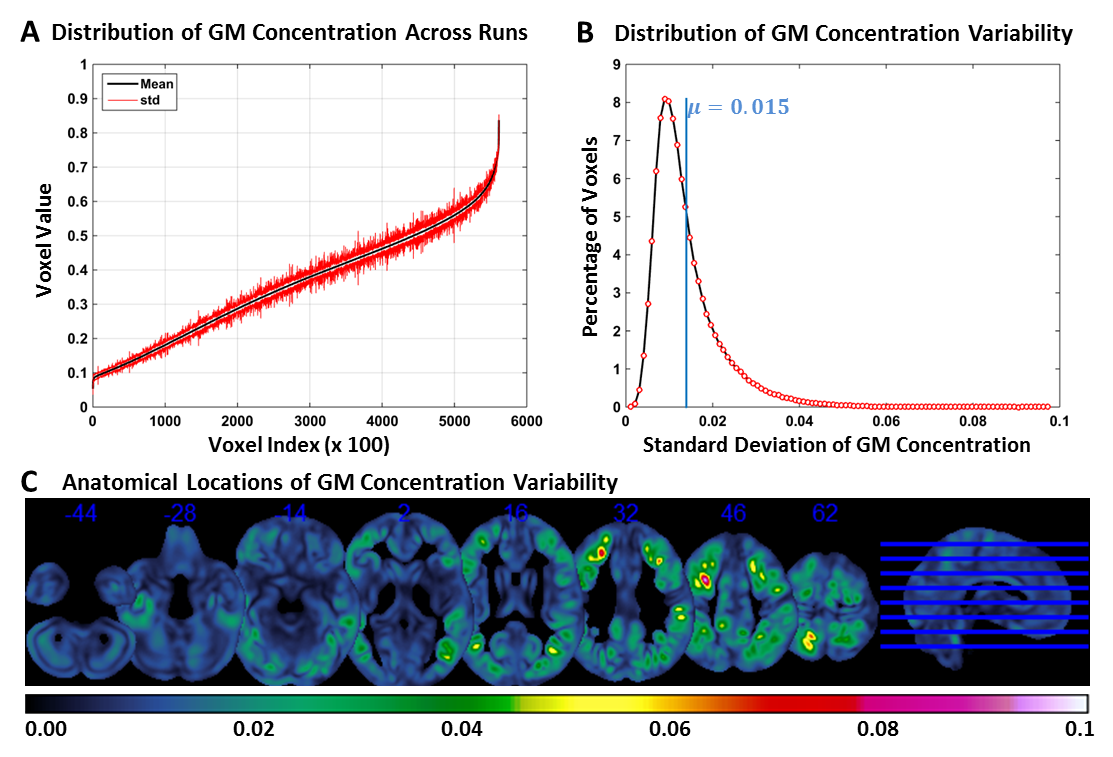


**Supp. Figure 2:** FINTM variability when preprocessed in groups with similar demographics (Experiment 1, constant subject 3).


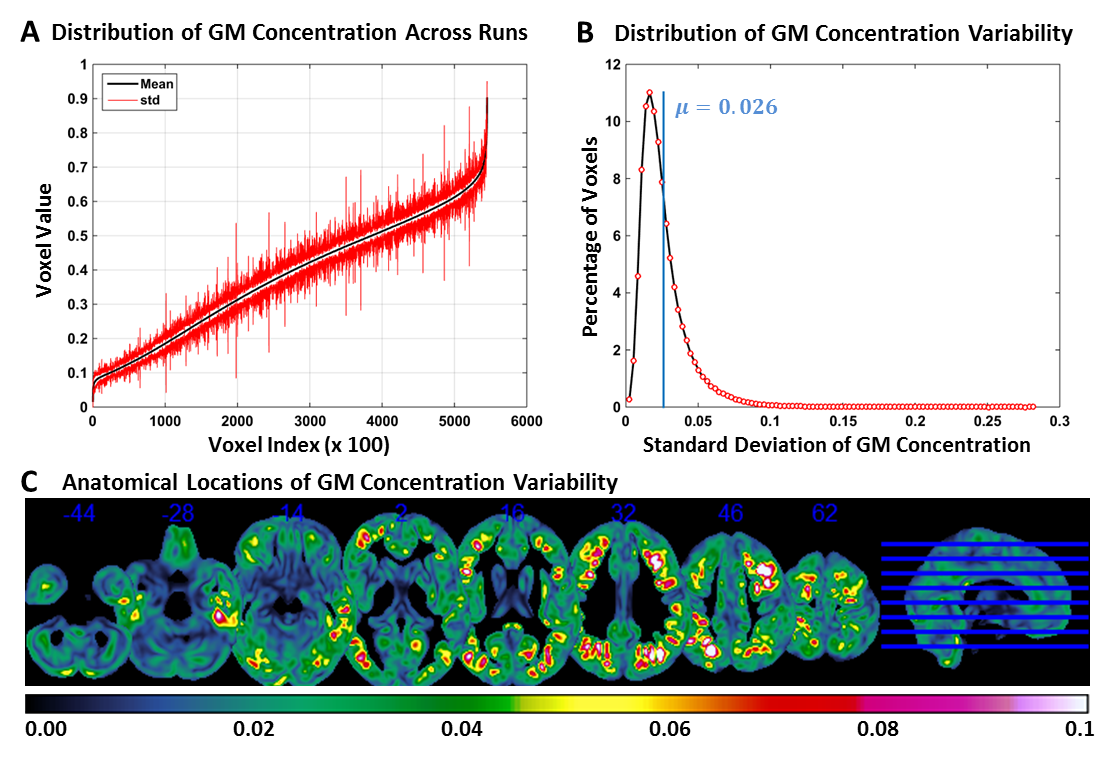


**Supp. Figure 3:** FINTM variability when preprocessed in groups with increasing age (Experiment 2, Constant Subject 2)


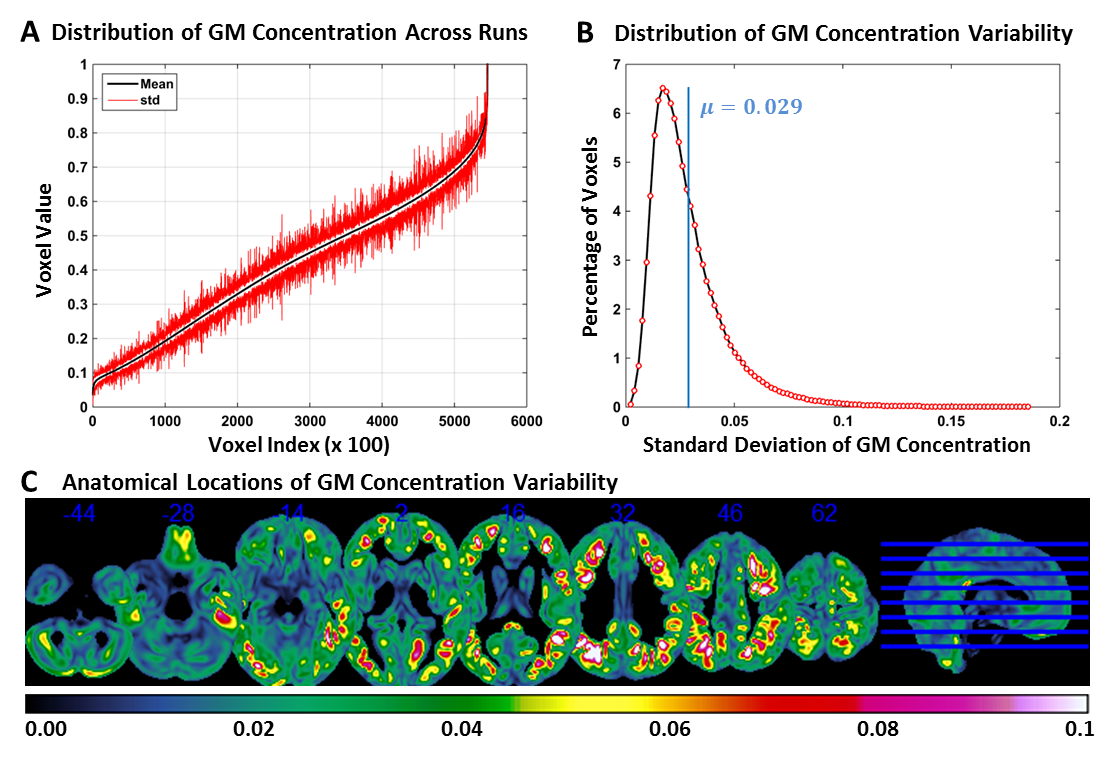


**Supp. Figure 4:** FINTM variability when preprocessed in groups with increasing age (Experiment 2, Constant Subject 3)


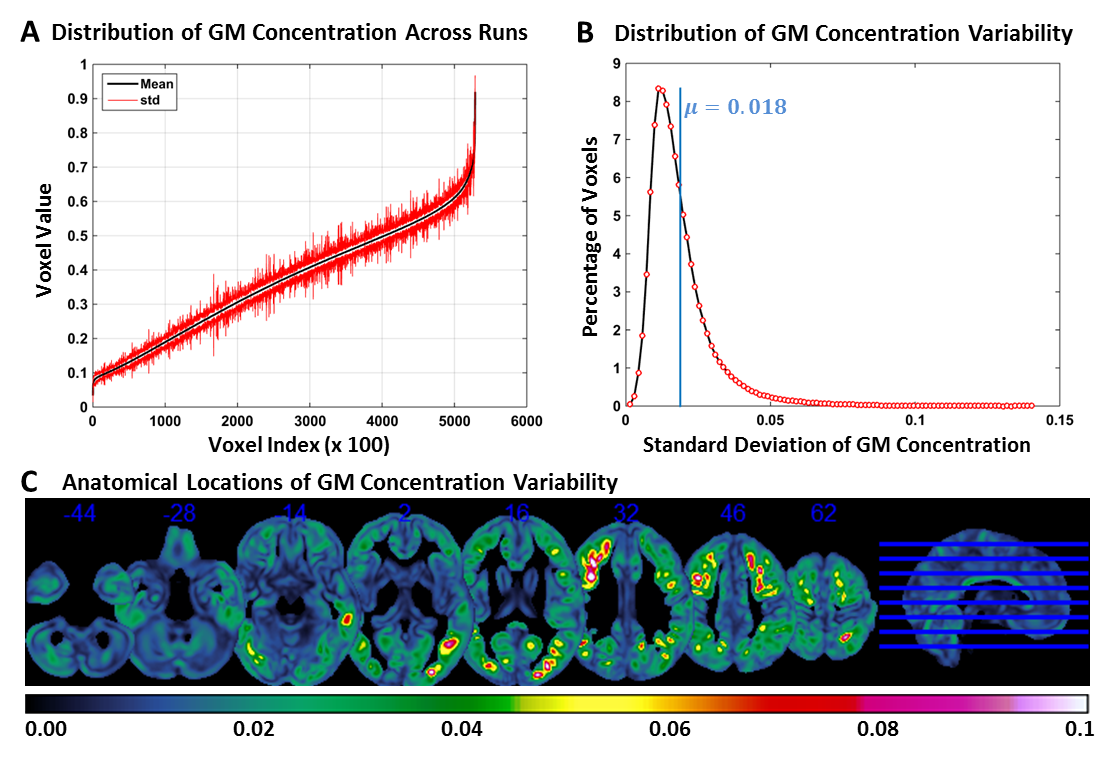


**Supp. Figure 5:** FINTM variability when preprocessed in groups with increasing ASD/TDC ration (Experiment 3, Constant Subject 2)


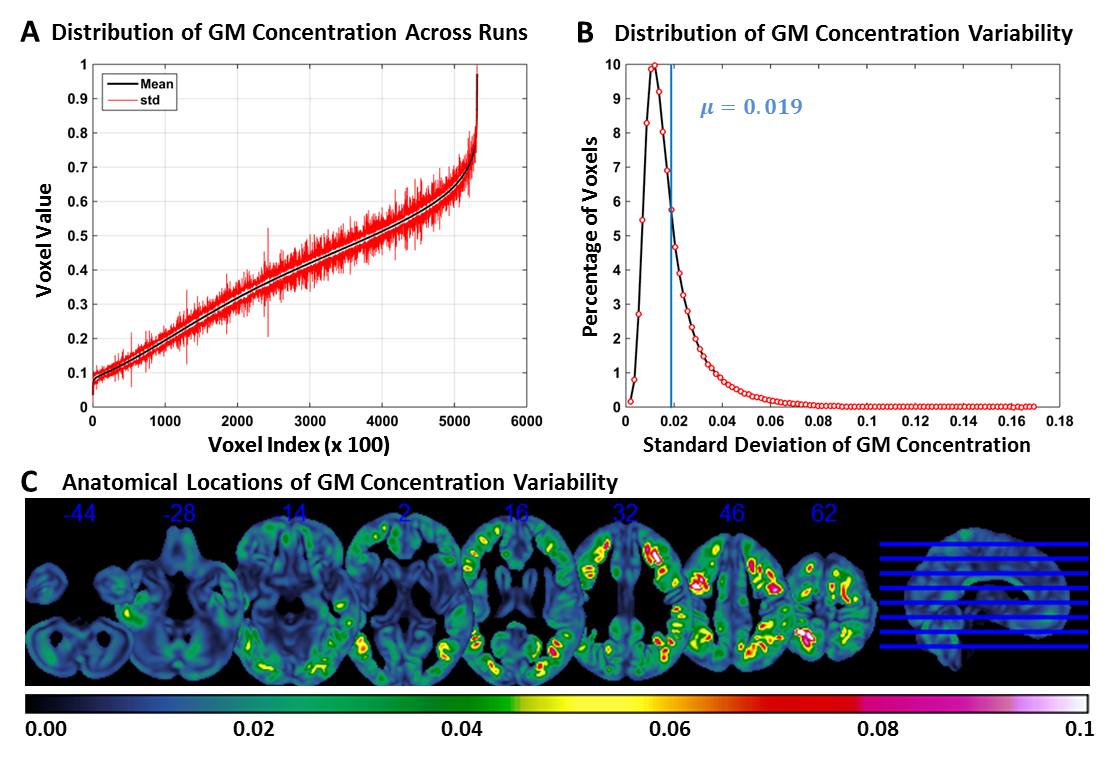


**Supp. Figure 6:** FINTM variability when preprocessed in groups with increasing ASD/TDC ration (Experiment 3, Constant Subject 3)
